# Mutation analysis of SDHB and SDHC: novel germline mutations in sporadic head and neck paraganglioma and familial paraganglioma and/or pheochromocytoma

**DOI:** 10.1186/1471-2350-7-1

**Published:** 2006-01-11

**Authors:** Jean-Pierre Bayley, Ivonne van Minderhout, Marjan M Weiss, Jeroen C Jansen, Peter HN Oomen, Fred H Menko, Barbara Pasini, Barbara Ferrando, Nora Wong, Lesley C Alpert, Rosie Williams, Edward Blair, Peter Devilee, Peter EM Taschner

**Affiliations:** 1 Department of Human Genetics, Leiden University Medical Center, Leiden, The Netherlands; 2 Department of Pathology, Leiden University Medical Center, Leiden, The Netherlands; 3 Department of Otorhinolaryngology, Leiden University Medical Center, Leiden, The Netherlands; 4 Department of Endocrinology, University Medical Center Groningen, Groningen, The Netherlands; 5 Department of Clinical Genetics and Human Genetics, VU University Medical Center, Amsterdam, The Netherlands; 6 Department of Genetics – Biochemistry and Biology, Turin University, Italy; 7 Cancer Prevention Centre, SMBD-Jewish General Hospital Montreal, Canada; 8 Department of Pathology, SMBD-Jewish General Hospital Montreal, Canada; 9 McGill University, Montreal, Canada; 10 Great Western Hospital, Swindon, UK; 11 Department of Clinical Genetics, Churchill Hospital, Oxford, UK

## Abstract

**Background:**

Germline mutations of the SDHD, SDHB and SDHC genes, encoding three of the four subunits of succinate dehydrogenase, are a major cause of hereditary paraganglioma and pheochromocytoma, and demonstrate that these genes are classic tumor suppressors. Succinate dehydrogenase is a heterotetrameric protein complex and a component of both the Krebs cycle and the mitochondrial respiratory chain (succinate:ubiquinone oxidoreductase or complex II).

**Methods:**

Using conformation sensitive gel electrophoresis (CSGE) and direct DNA sequencing to analyse genomic DNA from peripheral blood lymphocytes, here we describe the mutation analysis of the SDHB and SDHC genes in 37 patients with sporadic (i.e. no known family history) head and neck paraganglioma and five pheochromocytoma and/or paraganglioma families.

**Results:**

Two sporadic patients were found to have a SDHB splice site mutation in intron 4, c.423+1G>A, which produces a mis-spliced transcript with a 54 nucleotide deletion, resulting in an 18 amino acid in-frame deletion. A third patient was found to carry the c.214C>T (p.Arg72Cys) missense mutation in exon 4 of SDHC, which is situated in a highly conserved protein motif that constitutes the quinone-binding site of the succinate: ubiquinone oxidoreductase (SQR) complex in *E. coli*. Together with our previous results, we found 27 germline mutations of SDH genes in 95 cases (28%) of sporadic head and neck paraganglioma. In addition all index patients of five families showing hereditary pheochromocytoma-paraganglioma were found to carry germline mutations of SDHB: four of which were novel, c.343C>T (p.Arg115X), c.141G>A (p.Trp47X), c.281G>A (p.Arg94Lys), and c.653G>C (p.Trp218Ser), and one reported previously, c.136C>T, p.Arg46X.

**Conclusion:**

In conclusion, these data indicate that germline mutations of SDHB and SDHC play a minor role in sporadic head and neck paraganglioma and further underline the importance of germline SDHB mutations in cases of familial pheochromocytoma-paraganglioma.

## Background

Paragangliomas (PGL) of the head and neck (also known as glomus tumors) are parasympathetically innervated, benign tumors, most commonly arising at the carotid bifurcation, but also frequently found as vagal and jugulotympanic tumors. Pheochromocytomas are paragangliomas of the sympathetic nervous system, frequently catecholamine-secreting and arising in the chromaffin cells of the adrenal medulla. Extra-adrenal paragangliomas (often described as extra-adrenal pheochromocytomas) also originate from the sympathoadrenal neuroendocrine system and are designated based upon the primary anatomic site of origin.

Although many cases of head and neck paraganglioma are apparently sporadic (i.e. no known family history), clustering in families has long been recognized [1,2], and the search for susceptibility loci led to the mapping of two putative loci: PGL1-11q23 [2,3] and PGL2-11q13 [4]. The identification of PGL1 followed in 2000, when Baysal *et al *[5] reported germline mutations in SDHD (succinate dehydrogenase, subunit D) in PGL1-linked families. Subsequently, germline mutations in SDHC (PGL3-1q21) [6] and SDHB (PGL4-1p36) [7] were identified using a candidate gene approach, and both Astuti *et al *[7] and others [8-14] have reported that germline mutations of SDHB are involved in familial pheochromocytoma. It is now known that SDHB and SDHD, together with VHL and RET, play a major role in hereditary pheochromocytoma [15].

The three PGL genes identified to date encode subunits of the heterotetrameric succinate dehydrogenase complex of the mitochondrial-respiratory chain (complex II) and the Krebs cycle. While SDHB encodes one of the two subunits of the catalytic core, the iron-sulfur protein, the SDHC and SDHD proteins anchor complex II in the inner mitochondrial membrane.

Previously we reported the frequency of SDHD mutations in familial and sporadic head and neck paraganglioma in the Netherlands [16]. Nearly all clearly familial paraganglioma is accounted for by the Dutch founder mutations Asp92Tyr and Leu139Pro. In addition, about one third of sporadic cases are also due to one of these mutations. However, a group of 37 sporadic patients tested negative for SDHD mutations. Here we report the analysis of the SDHB and SDHC genes in this group of patients and in addition the analysis of 5 families presenting with pheochromocytoma and/or paraganglioma.

## Methods

### Patients

Patients ascertained in the Netherlands were from the Leiden University Medical Centre (Leiden, The Netherlands), the VU Medical Centre (Amsterdam, The Netherlands), the Utrecht Medical Centre (Utrecht, The Netherlands), and the University Medical Center Groningen (Groningen, The Netherlands). Other patients were ascertained from the Sir Mortimer B Davis-Jewish General Hospital, Montreal, Canada, the Instituto Nazionale Tumori, Milan, Italy and the Great Western Hospital, Swindon, UK. The institutional ethical committee of Leiden University Medical Center approved this study. All patients provided informed consent. Patients were diagnosed with head and neck paraganglioma, extra-adrenal paraganglioma or (adrenal) pheochromocytoma. In this article we subscribe to the tumor nomenclature described in Lack "Atlas of tumor pathology, Tumors of the Adrenal Gland and Extra-Adrenal Paraganglia" [17]. Cases were considered to be familial when one or more first or second-degree relatives were diagnosed with a related tumor, either paraganglioma or pheochromocytoma. Sporadic cases had no known family history. All studies were carried out using genomic DNA isolated from peripheral blood.

### Mutation analysis and sequencing

Mutation screening of the SDHB and SDHC genes was performed with genomic DNA and conformation sensitive gel electrophoresis (CSGE). The 8 exons of SDHB and the 6 exons of SDHC and at least 15–50 bp of each flanking intron were amplified (primers purchased from Sigma-Genosys, UK) (primer sequences available on request). Forward primers were labelled with FAM, HEX, or TET and amplified in a 10 μl reaction volume with 10 uM primers, 1× Super TAQ buffer (HT Biotechnology, UK), 0.6 mM dNTPs, and 0.02 U Silverstar *Taq *polymerase (EuroGentech, Belgium) and 100 ng genomic DNA sample. PCR was performed for 38 cycles (30 s; 94°C, 30 s; 53–62°C, 30 s; 72°C) with a final extension of 30 min at 72°C. Reaction mixtures were then pooled in a HEX:FAM:TET ratio of 3:2:2 and run on a CSGE gel with a modified gel matrix consisting of 0.5× MDE, 15% formamide in 1× TBE. The samples were run for 5.0 hours at 1680 V at 30°C on an ABI 377 DNA sequencer. Gels were analysed with GeneScan^® ^and Genotyper^® ^software (Applied Biosystems). Each abnormally migrating product was reamplified from the DNA sample using the same primers as above without label and sequenced in the forward direction using standard methods. All the patients ascertained in Leiden had previously been investigated for the presence of SDHD mutations using SSCP and restriction digestion analysis and found to be negative.

Mutations in three of the familial pheochromocytoma-paraganglioma cases were detected using CSGE analysis and confirmed by direct sequencing, while the remaining two were identified by direct sequencing of the SDHB gene, indicated by a suspicious clinical history. Further family members were identified as carriers using restriction analysis developed by us (Table 1).

**Table 1:** Variants/mutations of SDHB and SDHC with primers, restriction enzymes, PCR restriction products, and allele frequency.

**Variant**	**Gene**	**Primers^a^**	**PCR Product size**	**Enzyme**	**Normal Product**	**Variant Product**	**Allele^b ^Frequency**
Ser8Ser	SDHB	CGCGGCTAGTGGGTCCTCAG	165	Xho I	140+25	165	4/384
		CAAGGGTTGTGGCCGGCAACCGGCGCCTC**G**					
Ala6Ala	SDHB	CAGTGGATGTAGGCTGGGCGCC	130	Bsr BI	130	110+20	8/260
		AACCGGCGCCTCAAGGAG**C**G					
Trp47X	SDHB	TCCTTCAATAGCTGGCTTTCACAGA	119	Nco I	24 + 95	119	1/314
		ATCAAGAAATTTGCCATCTATC**C**ATG					
Arg94Lys	SDHB	GACTCTACTTTGACCTTCCGAAGATC**C**TGC	167	Pst I	109+33+25	134+33	0/296
		TAGGTTGCACAGCAAGTTCAC					
Arg115X	SDHB	ATCAATGGAGGCAACACTCTAGCTTGCA**G**C	150	Pvu II	150	125+25	0/402
		TGCAAATAAAAACAAAACCA					
c.423+1G>A	SDHB	CATGTATGTGATAAAGGATCTTGTTC**T**C	157	Bsp HI	157	132+25	0/294
		TTACTATCTGACTAGAAG					
Trp218Ser	SDHB	AGCTAATCATCCCTGGTTTT	215	Taq I	215	181+34	0/318
		TTGTGAGCACATGCTACTTC					
Arg72Cys	SDHC	GCCAATGAAACAGCCAAGTT	163	Dsa I (=Btg I)	49+22+92	49+114	0/328
		TTCCTTTTTAAAATTGTCTTTGTGTG					

^a^Mismatched nucleotides for the PIRA PCR technique are indicated in bold. ^b^Allele frequency is shown as total chromosomes.

Mutations are described in accordance with the recommendations of the Human Genome Variation Society (HGVS), update August 2004, and it should be noted that the current nomenclature can differ significantly from previous versions [18] and from that used in older literature.

### PCR screening of controls

Genomic DNA samples of 150–200 (300–400 chromosomes) healthy blood donors from the Leiden Blood Bank were screened for each of the mutations/polymorphisms. To detect SDHB variants, mismatch PCR primers were designed and appropriate restriction enzymes were selected using the PIRA PCR software [19] (Table 1). The SDHC variant could be detected by loss of a naturally occurring DsaI restriction site. PCR products were digested and visualized with ethidium bromide on agarose gels in the concentration ranges 2.0 to 3.0%

### RT-PCR analysis of splicing of SDHB (c.423+1G>A)

RNA was extracted from EBV-transformed PBLs using Trizol (Invitrogen, The Netherlands).

First strand synthesis of cDNA was carried out using 1 μg RNA, 4 ul of 5 × RT buffer, 5 mM dNTPs, 10 mM DTT, 100 units of MMLV RT and 1 μl of a 1:1 mixture of oligo dT/random hexamers (500 ng/μl) in 20 μl final volume at 37°C for 1 hour followed by PCR with 2 μl of the above reaction mixture and a forward primer for SDHB exon 3 (TGACTCTACTTTGACCTTCC) and a reverse primer for SDHB exon 5 (CTTCCTGAGATTCATCCTTC). The PCR reaction was as follows: 94°C, 3 min, with 38 cycles (94°C; 30 sec, 60°C; 30 sec, 72°C; 30 sec) followed by 5 min at 72°C. SDHB RT-PCR products were then analysed using 3% agarose gel electrophoresis/ethidium bromide and visualized on a UV illuminator.

## Results

Our study can be divided into two groups: 37 cases of sporadic head and neck paraganglioma from the Leiden University Medical Center; and 5 index cases with pheochromocytoma and/or paraganglioma and familial antecedents, collected from several other national and international clinical centers.

Of the 37 cases with sporadic head and neck paraganglioma, 2 individuals were identified as carrying heterozygous germline mutations of SDHB (5%).

Two patients carried a splice site mutation in intron 4, c.423+1G>A (Table 2). Patient S-003, a 50 year old male, presented with elevated catecholamine levels and single jugular paraganglioma, and died at the age of 58 due to complications resulting from tumor recurrence. A second male patient, S-020, presented at the age of 55 with a single carotid body tumor which was successfully removed, and a subsequent MRI scan three years later showed no abnormalities. This patient was described briefly in a previous report that examined SDH activity in paraganglioma, using the former mutation nomenclature of IVS4+1G>A [20]. The tumor from this patient was negative for SDH activity.

**Table 2:** Mutations of SDHB and SDHC found in this study.

**Gene Gene**	**Exon-Intron**	**Mutation^a^**	**Mutation (Protein)**	**Patient ID**	**Country of Origin**	**Age at Diagnosis**	**Sex (index case)**	**Family History**	**Clinical Features**
SDHB	exon 2	c.136C>T	p.Arg46X	FGT68	United Kingdom	32	Female	Sister and father (?)	Vagal and jugulotympanic paraganglioma
SDHB	exon 2	c.141G>A	p.Trp47X	FGT62	Italy	35	Female	Two sisters and paternal aunt, all carriers	Metastatic multifocal retroperitoneal paraganglioma
SDHB	exon 3	c.281G>A	p.Arg94Lys	FGT57	Pakistan	56	Male	Brothers and a nephew (proband and nephew tested and positive)	Retroperitoneal paraganglioma, jugulotympanic paraganglioma, adrenal tumor
SDHB	exon 4	c.343C>T	p.Arg115X	FGT61	Netherlands	36	Female	Sister and female maternal cousin	Bifocal extra-adrenal paraganglioma
SDHB	intron 4	c.423+1G>A	Splice Site	S-003	Netherlands	50	Male	Sporadic	Jugulotympanic paraganglioma
SDHB	intron 4	c.423+1G>A	Splice Site	S-020	Netherlands	55	Male	Unknown	Carotid body paraganglioma, unilateral
SDHB	exon 7	c. 653G>C	p.Trp218Ser	FGT66	Netherlands	68	Female	Daughter with paraganglioma	Extra-adrenal paraganglioma left para-aortal

SDHC	exon 4	c.214C>T	p.Arg72Cys	S-048	Turkey	36	Male	Sporadic	Carotid body paraganglioma, unilateral

^a^All mutations are novel except p.Arg46X. Mutations described in this report follow the recommended nomenclature of the HGVS, update August 2004.

**Figure 1:**
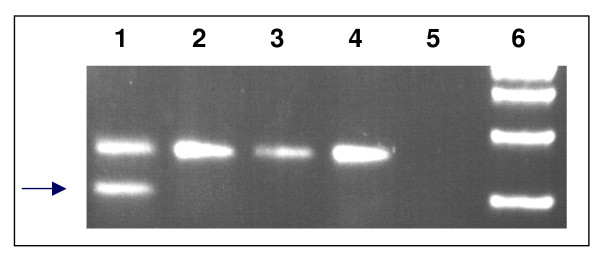
**The SDHB mutation c.423+1G>A disrupts normal splicing**. Primers in exon 3 and exon 5 were used to amplify cDNA. **Lane 1**: Patient S-020: In addition to a product of ~250 bp, a fragment missing 54 bp was also amplified (arrow). Sequencing showed an SDHB cDNA missing the proximal portion of exon 4. **Lanes 2–4**: RNA from three healthy controls included in the analysis revealed no additional PCR fragments, providing no evidence that the shorter product could be the result of normal alternative splicing. **Lane 5**: Water control. **Lane 6**: 100 bp ladder.

The splice site mutation in intron 4 abolishes the consensus splice donor sequence and thus may result in splicing abnormalities. RNA was available from patient S-020, and RT-PCR analysis confirmed that splicing was shifted 54 nucleotides upstream of the normal splice site, into the exon 4 coding sequence, leading to an in frame deletion of 18 amino acids (Figure 1). Normal control RNA did not reveal any alternative splicing. This variant was not found in the control population of 300 chromosomes. This mutation has recently been identified in two other patients of Dutch origin. As these patients are all of ethnic Dutch origin and are apparently unrelated, this mutation may represent a SDHB founder mutation in The Netherlands.

All sporadic patients were also analyzed for mutations in the six exons of SDHC. A single patient, a 36-year-old male of Turkish origin, was found to carry a germline mutation of SDHC. This patient was diagnosed with a single carotid body tumor, and had no known family history. A transition of C to T at base pair 214 in exon 4, resulted in the missense mutation of a highly conserved arginine residue (p.Arg72Cys), located in the putative quinone-binding site of complex II. A *C. elegans *mutant, *mev-1*, (SDHC) that results in oxidative stress and premature ageing also affects a residue of the quinone binding site, at position 69 of the human sequence [21]. This mutation is only the fifth identified in SDHC to date. The variant was not found in a Dutch control population of 328 chromosomes (although it should be noted that such a group is not an appropriate ethnic control to exclude the possibility of a non-functional Turkish polymorphism).

The functionality of all missense mutations should be interpreted cautiously, and particularly in a case such as this as this is only the second report of a missense mutation of SDHC and the patient presented without a family history.

In none of the sporadic cases was DNA available from the parents, so we were not able to establish whether these were *de novo *mutations.

In addition to the cases with sporadic head and neck paraganglioma, 5 index cases with pheochromocytoma and/or paraganglioma were collected, all of which were known or subsequently found to have a family history (Table 2).

The first index case, a 36-year-old Dutch female patient (FGT 61.1) presented with a suspected extra-adrenal paraganglioma, and two paragangliomas (close to left kidney) were identified. After surgical removal no additional clinical signs of paraganglioma could be detected. The sister of the index patient (FGT61.2) presented at the age of 27 with symptoms of dizziness and elevated noradrenalin. An extra-adrenal paraganglioma, located between the pancreas and left kidney was identified. A female cousin had previously been diagnosed with paraganglioma but was not available for testing. Both patients were found to carry a germline nonsense mutation of SDHB, c.343C>T (p.Arg115X) which leads to a truncation of the protein at under half its normal length of 280 amino acids.

A second Dutch index patient (FGT66), presented at the age of 67 (2004) with pain in the upper abdomen, and a CT-scan revealed a left-sided retroperitoneal mass. Normetanephrine was highly elevated and were no indications of localisations or metastases elsewhere. The patient's daughter had previously been diagnosed with paraganglioma of the carotid body, that was removed at the age of 29.

No other family members have clinical signs of either paraganglioma or pheochromocytoma. Analysis of DNA from the index patient revealed a missense mutation c. 653G>C (p.Trp218Ser) in exon 7 of SDHB. This missense change affects an amino acid that is conserved in both mammals and in *D. melanogaster*, *C. elegans*, *S. cerevisiae *and *A. thaliana*. This variant was not found in 318 chromosomes from healthy blood donors. The daughter of the index patient was subsequently tested and also carries the mutation. Other family members are currently receiving genetic counseling.

A 35-year-old Italian female (FGT62.1) presented with a family history of retroperitoneal paraganglioma, severe hypertension, and elevated urinary excretion of catecholamine metabolites. An extra-adrenal paraganglioma, infiltrating the Glissonian of the liver, was surgically removed. At follow-up, MIBG scintiscan showed 3 areas of intense uptake including the site of the previous surgery and two paravertebral lesions in the abdomen and chest. It is not known if these were metastases or primary tumors, as the patient refused further investigation. One year later the patient was suffering from widespread disease with bone metastases.

A sister (FGT62.2) of the index case presented at the age of 13 with an abdominal mass below the aortic bifurcation and increased urinary excretion of catecholamine metabolites. At surgery a 10 × 7 cm retroperitoneal paraganglioma was removed. At the age of 29, a left-sided cervical paraganglioma was diagnosed and removed. After two further relapses the patient presently shows bone metastases and a retroperitoneal mass, possibly a new tumor.

At the age of 60, a paternal aunt (FGT62.3) of the index case was found to have a paraganglioma of the posterior mediastinum. The patient had no further symptoms, and both she and her sons have refused further follow-up.

The index patient was found to carry a c.141G>A germline variant of SDHB resulting in a stop codon at codon 47 (p.Trp47X). Analysis of the DNA of the sister and aunt confirmed their carrier status for the mutation and demonstrated that the mutation segregated with disease. Surprisingly, this mutation was also identified in one sample from the Dutch blood donors screened as controls (1/314 chromosomes). As these donors are anonymous no information is available on the clinical status of this individual.

FGT57.6, a male of Pakistani descent, presented with a retroperitoneal paraganglioma, and had three male relatives (from a large extended family) with similar clinical profiles. A preoperative CT scan revealed a retroperitoneal mass adherent to inferior vena cava and extending up to the left renal vein. Pathology revealed a 9 × 8 × 4 cm encapsulated mass. This patient is currently doing well at age 61.

The brother (FGT57.8) of the index case was first seen at the age 47 with a paraganglioma arising in the diaphragm. The patient is currently alive. A second brother (FGT57.9) presented with adrenal cell carcinoma with bone and lung metastases. An arteriogram revealed an 11 cm highly vascularised adrenal neoplasm, most likely a primary adrenal, with vascular metastases to the left ileum. No remarkable hormonal changes were found. The patient died at age 41.

The nephew of the index patient (FGT57.7) presented at the age of 27 with a recent history of diminished hearing, pulsatile tinnitus and positional vertigo. A CT scan revealed a classic glomus jugulare tumour. The patient completed radiation therapy and remains stable.

The index case and his nephew were available for testing and both were found to be carrying a missense variant of SDHB, c.281G>A, resulting in the substitution of arginine for lysine at codon 94 (p.Arg94Lys). Arginine 94 is highly conserved and adjacent to a cysteine that constitutes one of the highly conserved cysteine regions that are the putative non-heme iron-sulfur clusters of SDHB [22].

Finally, a 32-year-old English woman (FGT68) presented with neck pain, swelling at the jaw, left vocal cord palsy and some wasting of the trapezius muscle. An MRI scan showed a para-pharyngeal mass, most likely a vagal paraganglioma. At surgery a highly vascular tumor virtually encircling the internal carotid artery and intimately connected to the hypoglossal nerve was revealed. A sub-total resection was carried out but the tumor progressed and the patient eventually died at the age of 39. The sister of the index patient underwent surgery for a jugulotympanic paraganglioma at the age of 33. The father of the index patient was still alive at the age of 75 and had a vague history of surgery for lumps on the face and neck. Other members of the family currently have no symptoms. The index patient was found to carry a previously described mutation in exon 2 of SDHB, c.136C>T, p.Arg46X [9]. The family is currently being offered genetic counseling.

## Discussion

Of the sporadic head and neck paraganglioma cases 8% were found to carry a germline mutation of SDHB or SDHC. Including our previous results [16], we have screened a total of 95 sporadic head and neck paraganglioma cases for germline mutations of SDH genes and found 24 (25%) in SDHD, 2 (2%) in SDHB and 1 (1%) in SDHC (total = 28%). Further studies will be required to discern whether the remaining cases can be explained by mutations of SDH genes not currently detectable with the techniques used or whether these are true sporadic cases, not attributable to germline mutations of one of the SDH genes.

In light of the known role of founder mutations in Dutch PGL cases [16], it seems likely that the majority of apparently 'sporadic' cases are related to other patients carrying the same mutation, rather than being true sporadic cases carrying *de novo *somatic or germline mutations. This seems particularly likely in the case of the very common SDHD mutation Asp92Tyr (D92Y) which accounts for 19 of the 24 sporadic SDHD cases, but may also explain the prevalence of the c.423+1G>A SDHB mutation.

Previous studies of mutations of SDH genes related to sporadic head and neck paraganglioma have examined SDHB, C, and D together [8,23-25] or SDHD only [16,26]. The four studies examining all three genes included 103 cases and found 3 mutations of SDHB, 0 mutations of SDHC and 7 mutations of SDHD. Including the current study, a total of 5 mutations of SDHB have been found in 141 cases (3.5%), and 1 of SDHC (0.7%). The two studies that looked only at SDHD were both conducted in The Netherlands and found 33 mutations in 93 Dutch cases (35%). The higher incidence in The Netherlands is largely attributable to the common Dutch mutations Asp92Tyr (D92Y), Leu95Pro (L95P) and Leu139Pro (L139P).

While this study was conducted with the aim of identifying the incidence of germline mutations of SDHB and SDHC in paraganglioma/pheochromocytoma, it is worth remembering that we did not examine DNA from tumors, so no conclusion can be drawn on the incidence of somatic mutations of SDHB and SDHC in paraganglioma. Previous studies that did address this question, including SDHD, [7-9,25-30] found no evidence of somatic SDH mutations playing a role in paraganglioma, with the exception of Gimm *et al *[31] who reported a single case with a somatic mutation of SDHD, Pro81Leu. This mutation has since been commonly reported in germline samples of individuals of Northern European origin, but has never again been found only in tumor DNA.

The five novel SDHB mutations described here further underline the importance of germline mutations of this gene in cases of paraganglioma and pheochromocytoma.

The striking variability in the penetrance and phenotype of SDHB mutations in several the families described illustrates the difficulty in ascertaining the family history of patients. Similarly, McDonnell *et al *[11] recently described a remarkable pedigree in which a single case, a young boy, led to the detection of a further 17 SDHB mutation carriers, 4 of whom were phenotypically affected.

The true contribution of heredity in the incidence of paraganglioma/pheochromocytoma is currently undergoing a reappraisal [32]. The traditional view of pheochromocytoma, in which 10% of tumors were thought to be hereditary [33] has now been superceded by the realization that germline mutations underlie at least 25% of cases [12]. Much of this increase is attributable to the recent availability of genetic testing of SDH genes. The full extent of the role of germline mutations may still have been underestimated. When the incidence of clinically manifest pheochromocytoma (1 in 500,000) [34-36] is compared to that found in two autopsy studies (1 in 2000) [37,38], it seems that only a minority of tumors are being detected clinically. Even considering an ascertainment bias of the autopsy studies due to unrecognized pheochromocytoma-related cause of death, the difference in prevalence suggests that many pheochromocytoma patients without or with mild symptoms do not seek medical help and thus will not come to a clinician's attention.

This may have important implications for both the general biology and genetics of these tumors. A full understanding of the biology of adrenal and extra-adrenal paragangliomas in relation to paragangliomas of the parasympathetic system, both in regard to the spatial limitations for undetected tumor growth in the head and neck region compared to the abdomen, and the extent to which adrenal and extra-adrenal paragangliomas produce catecholamines, might eventually lead to some convergence in the phenotypic description of these tumors.

A recent study of patients with SDHD-linked head and neck paragangliomas [39] showed that both catecholamine excess and extra-adrenal paragangliomas/pheochromocytomas in this group are higher than previously thought, suggesting that SDH related tumors show distinct [13] but still broadly overlapping phenotypes.

The patient carrying a SDHC mutation described in this report joins the small number of individuals with SDHC mutations reported to date, which include a family showing five cases of non-chromaffin paraganglioma and carrying a mutation that ablates the start codon ATG, (c.3G>A) [6]. A subsequent report described a sporadic female patient presenting with catecholamine-secreting carotid body tumor, showing metastasis to a lymph node. A splice donor site mutation (IVS5+1G>A) was identified in this patient, resulting in the skipping of exon 5 [40]. More recently Bauters *et al *[41] identified a missense mutation (c.473T>C; Leu158Pro) in a case of carotid body paraganglioma, and Baysal *et al *[42] described a family with head and neck paraganglioma with an 8 kb Alu-mediated SDHC deletion. The patient described in this report, a 36-year-old male of Turkish origin, was diagnosed with a single carotid body tumor (3.5 cm diameter), and no other complications.

The c.214C>T change in SDHC results in the substitution of arginine for cysteine. Arginine 72 (equivalent residue in *E. coli*: – Arg31) is situated in a highly conserved protein motif (E. *coli*: aa27-SILHR-aa31, *S. cerevisiae*: aa43-SSLHR-aa47, *H. sapiens*: aa68-SICHR-aa72) that constitutes the quinone-binding site of the succinate-ubiquinone reductase (SQR) complex in *E. coli*, [43,44]. Mutants of *E. coli *carrying a substitution of arginine 31 are unable to grow aerobically on succinate as a carbon source. In addition, mutation of the histidine adjacent to Arginine 47 caused a marked reduction in the catalytic efficiency of quinone reduction in *S. cerevisiae *[45].

Although relatively few SDHC mutations carriers are known, totaling 15 individuals, including 4 with no other known affected family members, all affected individuals had head and neck paragangliomas, including one case of a metastatic, catecholamine-secreting carotid body tumor. Despite the small numbers the parallels with SDHD are obvious, and not altogether surprising in the light of the probable functional equivalence of these genes.

## Conclusion

In this study we detected germline mutations of SDHB in 5% and of SDHC in 2.5% of sporadic head and neck paraganglioma cases. Including data from published studies, a total of 5 mutations of SDHB (3.5%) and 1 of SDHC (0.7%) have been reported in 141 cases. Together with our previous study of SDHD mutations we have now found 27 SDH germline mutations in 95 cases (28%) of sporadic head and neck paraganglioma, the majority of these being Dutch founder mutations of SDHD. These data indicate that germline mutations of SDHB and SDHC play a minor role in sporadic head and neck paraganglioma. In contrast, germline mutations of SDHB were found in all cases of familial pheochromocytoma and/or paraganglioma. The clinical data presented illustrates the striking variability in phenotype related to SDHB mutations, even within a single family.

The mutation of SDHC reported here is only the fifth to be described, extending our knowledge of the phenotype related to mutations of this gene

## Competing interests

The author(s) declare that they have no competing interests

## Authors' contributions

JPB collected, edited and analysed the data, co-designed the study, and wrote the manuscript. PEMT and PD designed and implemented the study, and contributed to the manuscript. IvM collected and analysed the data. MMW, JCJ, LCA, BF, RW, EB collected and edited the data. PHNO, FHM, BP, and NW collected and edited the data and contributed to the manuscript.

## Pre-publication history

The pre-publication history for this paper can be accessed here:


